# Long-distance trade in the Middle Chalcolithic of the southern Levant: The case of the olivine beads from Tel Tsaf, Jordan Valley, Israel

**DOI:** 10.1371/journal.pone.0271547

**Published:** 2022-08-10

**Authors:** Danny Rosenberg, Yael Elkayam, Yossi Garfinkel, Florian Klimscha, Vesna Vučković, Yaakov Weiss

**Affiliations:** 1 Laboratory for Ground Stone Tools Research, Zinman Institute of Archaeology, University of Haifa, Haifa, Israel; 2 The Martin (Szusz) Department of Land of Israel Studies and Archaeology, Archaeogemology Lab., Bar-Ilan University, Ramt Gan, Israel; 3 Institute of Archaeology, The Hebrew University of Jerusalem, Mount Scopus, Jerusalem, Israel; 4 Lower Saxony State Museum, Department of Research/Collections, Archaeology Division, Hanover, Germany; 5 Institute of Earth Sciences, The Hebrew University of Jerusalem, Jerusalem, Israel; Universita degli Studi di Milano, ITALY

## Abstract

Eight olivine beads found at the Middle Chalcolithic site of Tel Tsaf (ca. 5,200–4,700 cal. BC), Jordan Valley, Israel, underscore a new facet of interregional exchange for this period. The current paper presents the olivine beads assemblage, its morphometric and technological characteristics, and chemical composition. The results of the chemical analysis suggest that all eight beads derive from the same source. By means of comparison with the chemical characteristics of known olivine sources, we argue for a northeastern African–western Arabian provenience and cautiously suggest Ethiopia as a probable origin. Finally, we discuss the significance of the assemblage, its possible origin, and the mechanisms that may have brought the beads to the site.

## 1. Introduction

In antiquity, beads and other items of personal adornment appreciated by the community were among the principal devices for transmitting social and economic information over large distances. By token of their small size, they could change hands easily, while their aesthetic and symbolic value rendered them economically effective [1]. Notably, beads fulfill various functions [1–4] and are often considered markers of identity, social status, and even economic progress and stability [5,6].

In the prehistoric southern Levant, exchange and trade in beads was common practice for more than a hundred thousand years [7,8], and while most were made from locally available raw materials, exotic rocks imported from farther away were also recorded (e.g., obsidian, turquoise, amazonite, amethyst, serpentine [7,9–11]).

Among these exotic minerals, olivine (sometimes called *peridot or chrysolite* [12–14]) is one of the rarest. This mineral is common in igneous rocks, measuring 6.5–7 on the Mohs hardness scale, and is usually yellow-green or green in color. Chemically, olivine is a continuous solid solution between the end-members forsterite (Fo; Mg_2_SiO_4_) and fayalite (Fa; Fe_2_SiO_4_). It is the most common mineral in the Earth’s mantle and the first mineral to crystallize in the basaltic magmas. Consequently, large olivine crystals are uncommon on the Earth’s surface and are restricted to slowly cooled mantle rocks: xenoliths or ophiolites. In basalts, olivine phenocrysts are commonly small (<2 mm in size [15,16]). Notably, unlike other igneous gem-minerals (e.g., garnet, tourmaline, beryl), olivine is characterized by low concentrations of most trace elements (well below 1 ppm), which do not fit well its simple crystal structure and chemical composition [17–19].

Viewed from the southern Levant, the nearest olivine sources with sufficiently large crystals to facilitate bead production must be sought in remote locations. Casting a wide net over southwestern Asia, northeastern Africa, and Arabia, we may speak of six possible sources: (1) Egypt, specifically Zabargad (St. John’s Islands) in the Red Sea [20–23] and the Eastern Desert [24]; (2) Harrat Kishb, Saudi Arabia [25–28]; (3) southwest and northwest Turkey [29–31]; (4) Kohistan, northwest Pakistan [32–34]; (5) the Ethiopian plateau and main Ethiopian rift area [35–37]; and (6) the north Tanzanian divergence area [38–40].

Under these circumstances, it is unsurprising that the occurrence of olivine in the southern Levant is scarce. The earliest documented case in the region for using this mineral for beads and other artifacts (such as pendants and emulates) is from Predynastic Egypt [20,41,42]. At a much smaller scale, a bead from the Late Chalcolithic (ca. 4,500–3,900 cal. BC) burial cave of Peqi’in, Israel, was provisionally identified as olivine [10], and a Bronze Age neckless made of olivine beads was found in Raqqa, Syria [43].

Archaeometric and microscopic research on beads has developed considerably in the last few years, providing new tools and methodologies for investigating exchange networks, technical innovations, and cultural and political contacts. Thus, the significance of these venues of analysis lies in their ability to reconstruct bead movements across the landscape and modify how we interpret social interactions. This paper discusses a corpus of olivine beads from the Middle Chalcolithic site of Tel Tsaf, the Jordan Valley, Israel. Roughly dated to the late-sixth–early-fifth millennium cal. BC, Tel Tsaf constitutes the earliest occurrence of olivine artifacts in the southern Levant. Below, following a brief introduction to the site of Tel Tsaf, we describe the olivine beads, reconstruct the procedures of their production, set out to determine their chemical attributes, and trace their origin. Finally, we discuss the significance of their presence at the site and its possible implications.

## 2. Materials: Tel Tsaf and the olivine beads assemblage

**Fig 1:**
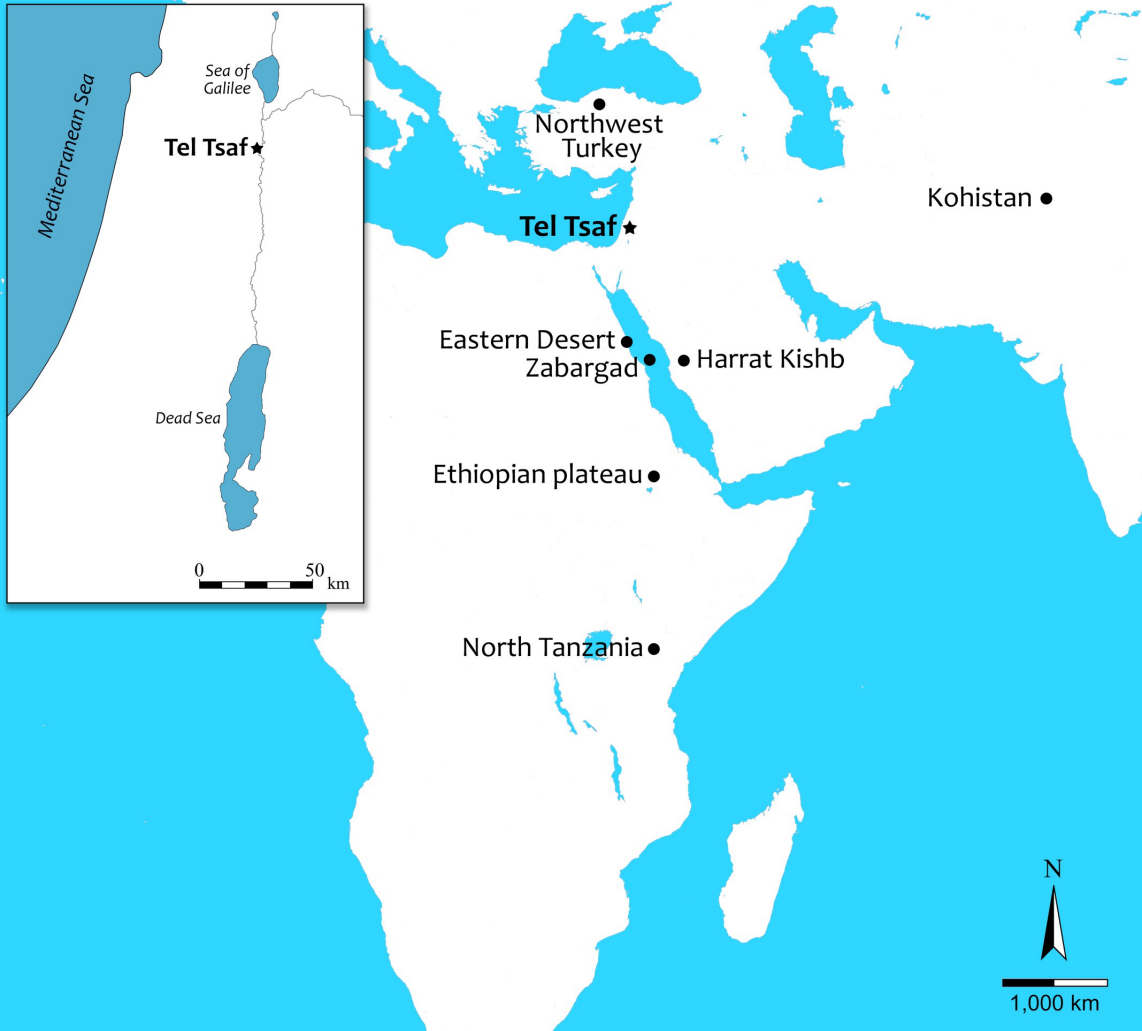
A map of the Near East and Africa with the locations of potential olivine sources (created by S. Haad).

**Fig 2:**
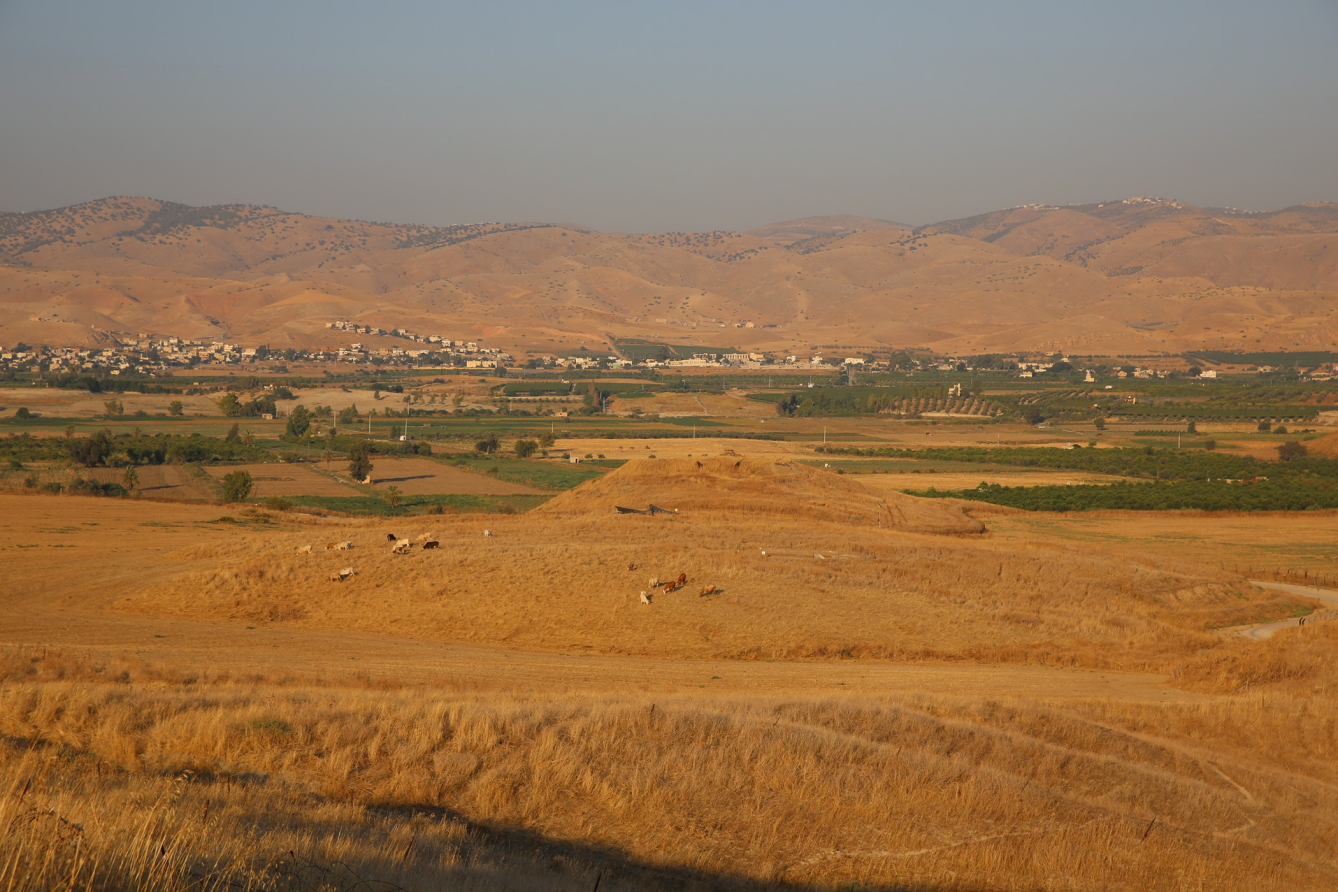
Tel Tsaf. A view from the west.

Tel Tsaf is a ca. 5 ha site in the Middle Jordan Valley, Israel (Figs 1 and 2), comprising three hills and their immediate surroundings [44,45]. The site was first recorded in the 1940s [46] and excavated by three expeditions. The first occurred between 1977 and 1980 [47] and the second between 2004 and 2007 [44,48]. The third expedition was initiated in 2013 and is still ongoing. It focuses on various aspects of long-distance contact, site economy and the establishment of the Mediterranean diet in the region, social organization, and ecology [11,49–52].

Tel Tsaf was settled during ca. 5,200–4,700 cal. BC [50,53] and densely occupied throughout. Excavations revealed an intricate assortment of structures and installations, including courtyard buildings [44], silos, and roasting pits, probably reflecting large-scale storage and feasting [44,48,51,52,54–56]. The structures were built of sun-dried mudbricks, and their walls were coated with plaster [56]. Large quantities of diverse faunal (consisting mainly of domesticated animals [57]) and floral remains were found (e.g., seeds, phytoliths, pollen, starch granules [50,58]) in association with storage, cooking, and roasting installations. Notably, the site seems to provide evidence for the crystallization and establishment of the Mediterranean diet [11,49,52], including the gradually increasing significance of olives alongside the use of dairy products [59].

Tel Tsaf is also notable for its numerous non-local exotic finds, indicating the site’s participation in a far-reaching exchange network [11,52]. A partial list includes beads from Transjordan, beads and Nilotic shells from Egypt, tokens, and figurines from the northern Levant, obsidian from Anatolia, a copper awl from an indeterminate northern origin [60], Ubaid style pottery from Mesopotamia, and seashells from the Mediterranean coast.

Specifically concerning the occurrence of beads at the site, a massive assemblage of over 2,500 ostrich eggshell beads is notable [44]. Otherwise, dozens of beads were also recoded; many were made from non-local rocks and minerals, while others were produced from clay, bone, wood, and *Theodoxus Jordani* shells [61,62].

**Fig 3:**
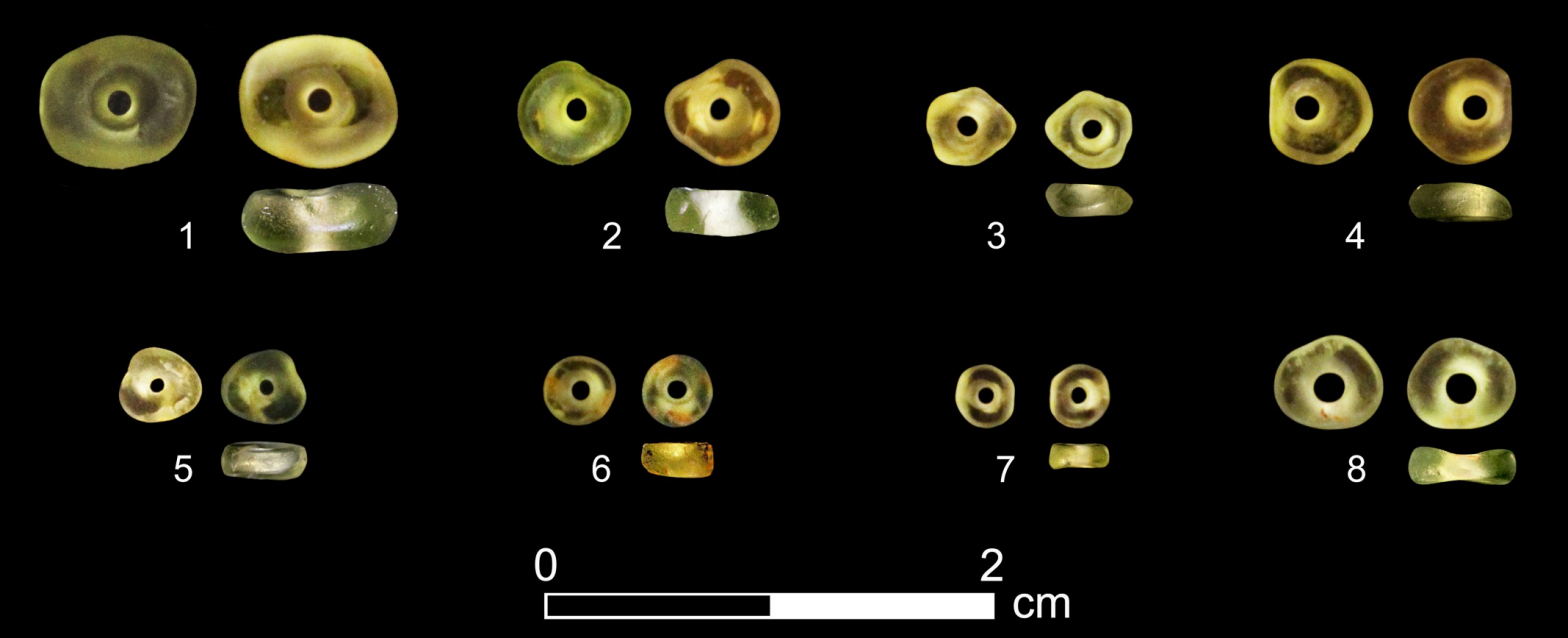
The Tel Tsaf olivine beads.

So far, excavations at Tel Tsaf have produced eight olivine beads retrieved from various contexts in Area C (Fig 3; Tables 1 and 2). Seven were found during the current excavation project and one during the 2004–2007 expedition. Six were retrieved from Square AR16, a deep cut in Area C. Of these, one was in a pit, and the others were in disparate accumulations. Of the remainder, one bead was recovered from accumulations north of Room C70 in Building complex CI, and the other was found in fills of a Byzantine/Early Islamic grave.

**Table 1:** The context of the Tel Tsaf olivine beads.

Item	Area	Locus	Basket	Context	Figure
Tsaf-Olivine1	C	2066	3355	Accumulations in Sq. AR16	3:1
Tsaf-Olivine2	C	2274	4400	A pit in Sq. AR16	3:2
Tsaf-Olivine3	C	2184	3940	Accumulations in Sq. AR16	3:3
Tsaf-Olivine4	C	907	2213	Accumulations north of C70	3:4
Tsaf-Olivine5	C	2020	3044	Accumulations in Sq. AR16	3:5
Tsaf-Olivine6	C	2196	4042	Accumulations in Sq. AR16	3:6
Tsaf-Olivine7	C	1011	2816	Accumulations in Sq. AR16	3:7
Tsaf-Olivine8	C	264	595	Fills (containing Middle Chalcolithic remains) of a Byzantine/early Islamic grave cut into the Middle Chalcolithic layers	3:8

**Table 2:** The characteristics and properties of the olivine beads.

Bead	Shape	Profile	Weight (g)	Minimum measurement across (mm)	Maximum measurement across (mm)	Thickness (mm)	Drilling	Perforation minimum diameter (mm)	Perforation max diameter of side A (mm)	Perforation max diameter of side B (mm)
Tsaf-Olivine1	oval	oval	0.2	6.0	8.0	3.0	+	0.4	1.0	1.0
Tsaf-Olivine2	oval	oval	0.08	5.0	6.0	2.0	+	0.4	0.9	1.0
Tsaf-Olivine3	oval	oval	0.03	4.0	5.0	1.0	+	0.3	0.6	0.7
Tsaf-Olivine4	triangle	oval	0.064	5.0	5.0	2.0	+	0.6	0.7	1.0
Tsaf-Olivine5	triangle	oval	0.032	5.0	4.0	2.0	+	0.3	0.5	0.5
Tsaf-Olivine6	round	oval	0.033	3.0	3.0	2.0	?	0.3	0.6	1.0
Tsaf-Olivine7	round	oval	0.015	3.0	3.0	2.0	?	0.3	0.5	0.6
Tsaf-Olivine8	oval	oval	0.05	4.0	4.0	2.0	?	0.5	0.8	0.9

All eight beads are whole and characterized by a dark to light green color with a tint of yellow hue (Fig 3). Optical examination indicated that all beads are translucent and lack foreign inclusions. They have an oval-lenticular cross-section, and their shape varies from round, through oval, to quasi-triangular. The beads’ perforations are biconical, and their two faces are sometimes even.

The olivine beads weigh 0.015–0.2 g and are 1.0–3.0 mm thick. Their smallest measurements across are 3.0–6.0 mm, while their maximum measurements are 3.0–8.0 mm. All perforations have round outlines (Fig 3) and seem to have been drilled from both sides (bidirectional drilling). Their minimum diameter is 0.3–0.6 mm, and their maximum diameter ranges between 0.5 and 1.0 mm across (for both faces).

## 3. Methods

All beads underwent attribute analysis at the Laboratory for Ground Stone Tools Research (LGSTR) at the Zinman Institute of Archaeology, University of Haifa. First, the beads’ morphometric and contextual properties were documented, including circumstances of discovery, degree of preservation, shape, measurements, color, and profile. Next, aided by a Dino-light, edge 3.0, digital microscope (magnification 10–150×), microwear patterns indicative of production and use were recorded.

At the Institute of Earth Sciences at the Hebrew University of Jerusalem, the beads were subjected to further optical examination with Hirox RH-2000 and Nikon SMZ800 binocular microscopes. Preparing for geochemical analyses, we immersed the beads in epoxy and mildly polished 1 μm off one side, using diamond polishing powder. We completed the treatment by cleaning the beads with ethanol and distilled water in an ultrasonic bath.

All specimens were subjected to Raman spectroscopic analysis at the Center for Nanoscience and Nanotechnology, the Hebrew University of Jerusalem, to determine the beads’ mineralogical compositions. We used a Renishaw InViaTM Raman microscope, equipped with Argon-Ion Laser 50 mW (514 nm) and a Leica DM2500-M microscope. Each spectrum was acquired for 10 seconds of illumination, using a 20× objective and the Renishaw CCD camera (1040×256 px). Laser power for all analyses was set at 5%, and spectra were collected between 100 and 1500 cm^-1^. The spectral resolution was 1 cm^−1^, and the spectrometer was calibrated against the 520 cm^−1^ peak of a synthetic silicon plate. Mineral identification was made against spectra of the Hebrew University mineral collection and the online RRUFF™ project spectral data for minerals (https://rruff.info/).

The beads were also subjected to elemental analysis at the Institute of Earth Sciences at the Hebrew University of Jerusalem. We employed a JEOL JXA-8230 electron probe microanalyzer (EPMA) equipped with four wavelength dispersive spectrometers. Across all analyses, the electron beam used was 1 μm in diameter with an acceleration voltage of 15 kV and a current of 120 nA. Peak counting rates were 30 s for the major elements (Si, Mg, and Fe) and 180 s for minor elements (Ca, Ni, Cr, Mn, and Al), while background counting rates were half the time for each background position (i.e., 15 s and 90 s, respectively). A set of reference materials were used to calibrate the EPMA for specifiable elements: San-Carlos olivine (NMNH# 111312–44) for Si, Mg, and Fe, SPI standard diopside (MgCaSi_2_O_6_) for Ca, chromite (FeCr_2_O_4_) for Cr, rhodonite (MnSiO_3_) for Mn, pentlandite ((Fe, Ni)_9_S_8_) for Ni, and an in house HUJI spinel (MgAl_2_O_4_) for Al. These standards were analyzed as unknowns for calibration control and to monitor instrument stability. The ±2σ analytical uncertainty of 75 analyses of San Carlos olivine produced ±0.46 Fo# [where Fo# is the molar ratio of 100Mg/(Mg+Fe)], ±158 ppm Ni, ±41 ppm Ca, ±127 ppm Mn, ±18 ppm Al and ±24 ppm Cr.

## 4. Results

### 4.1 Microwear traces and the production sequence

**Fig 4:**
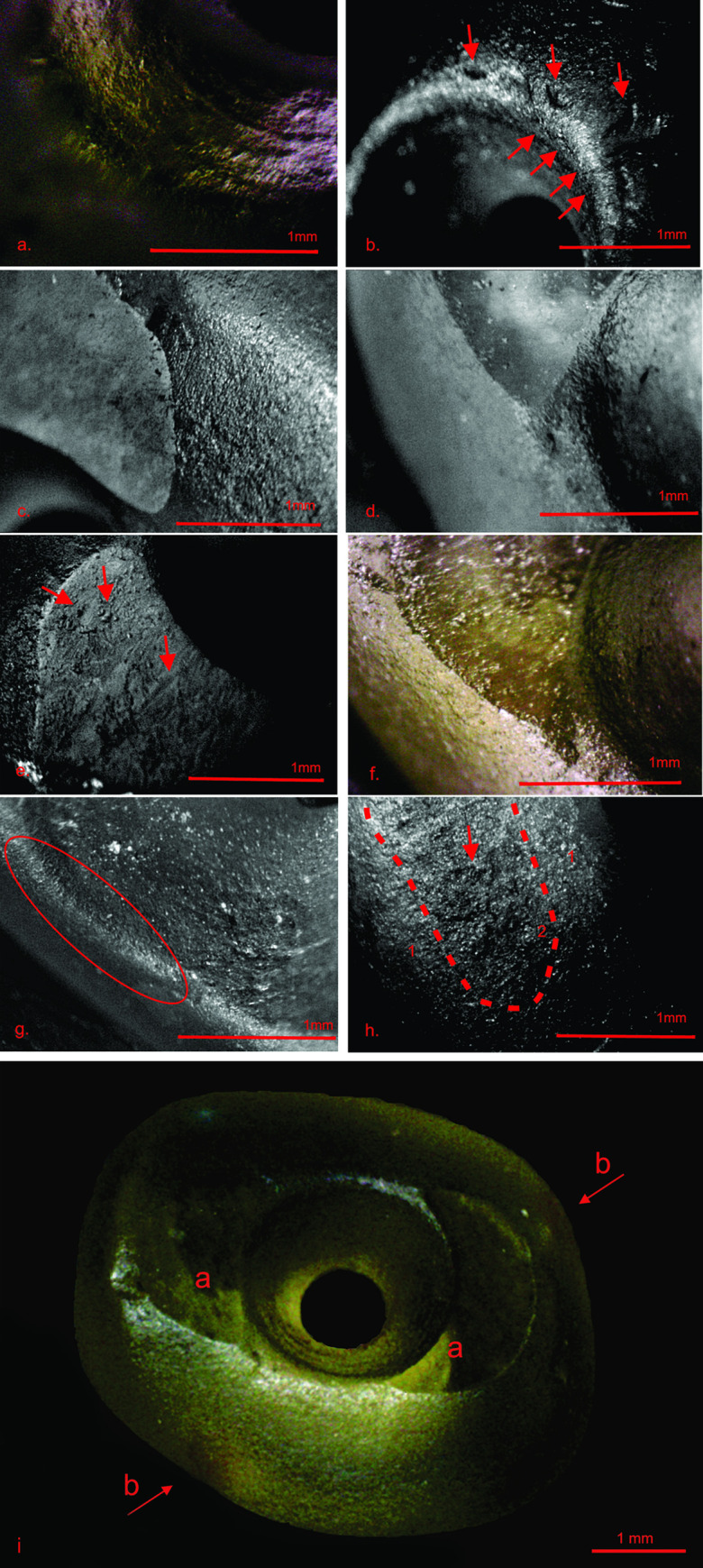
Micro use-wear traces on the Tel Tsaf olivine beads. ) a) A perforation’s wall bearing micro-scratches vertically and horizontally aligned to the bead’s main axis; (b) A micro-flake scar (arrow) attributed to pecking and micro-scratches (arrow) perpendicular to the perforation’s rim; (c) Micro-scratches and traces of pecking along the edge of the even surface; (d) Micro-scratches on an area between the perforation’s rim and the even surface; (e–f) Micro-pits, micro-flake scars, and micro-scratches on the even surface (arrow); (g) Micro-scratches on the perforation’s rim; (h) A smoothed surface with rounded grains and a low microtopography (1) alongside traces of pecking (arrow) and wear (2). (i) Platforms: Shiny, leveled surfaces (a) and red particles on Tel Tsaf olivine beads (b).

We observed four types of microwear traces on the beads: scratches, flake scars, pits, and edge rounding. Micro-scratches are widespread and observed on practically all parts of the bead: perforation walls (including both horizontal and vertical scratches; Fig 4A), perforation rims (Fig 4B and 4G), leveled surfaces (Fig 4E and 4F), leveled surfaces’ circumferences (Fig 4C), and areas between the perforations and leveled surfaces (Fig 4D). Slightly less extensive micro-flake scars were recorded along beads’ perforation rims (Fig 4B), the leveled surfaces (Fig 4E), and the leveled surfaces’ circumferences (Fig 4C). Lastly, edge rounding (Fig 4H) and micro-pits (Fig 4E and 4F) were observed on leveled surfaces only (Fig 4I).

Drawing on these observations, we may carefully infer the beads’ *chaînes opératoires*. We presume that the scratches were produced by grinding with an abrasive powder [63], whereas micro-flake scars were created by percussion. These would have been the first operations applied to the olivine crystals. Next, the beads’ leveled surfaces were produced, removing most abrasion and flake scars, although some remained along the edges (Fig 4C, 4D and 4F). While these surfaces (Fig 4A) seem aesthetically desirable, they could have served a technical function as drill-facilitating platforms [64]. The surfaces’ shine might suggest that heat treatment was also applied. Such treatment has been shown to render siliceous matrixes finer [65,66] and a stone’s color more intense without altering its translucence or hardness. Further support for heat application in the beads’ production process is provided by the observation of luster in fresh breaks of platforms (Fig 4I: a) [65] and the occurrence of intensive red particles in some beads’ fabrics (Figs 3: 5–6, 3: 8 and 4I: b).

The beads’ perforation was carried out by drilling from both sides, producing a biconical shaft. This procedure was employed to avoid damage or breakage of the bead. The drill was probably made of a hard stone such as flint or quartz [64,67,68], and its application presumably entailed the use of a lubricant [63,69] (Fig 4A).

The last step of the *chaîne opératoire* was smoothing a bead’s surfaces (Fig 4E). The rounded grains and low micro-topography observed on these surfaces suggest that a soft material was used, possibly wood or linen [63,64]. Abrasive dust produced in the grinding stage of the manufacturing process might have also been used for smoothing, a possibility suggested by ethnographic and experimental observations [64,70,71].

### 4.2 Raw material and chemical attributes

**Fig 5:**
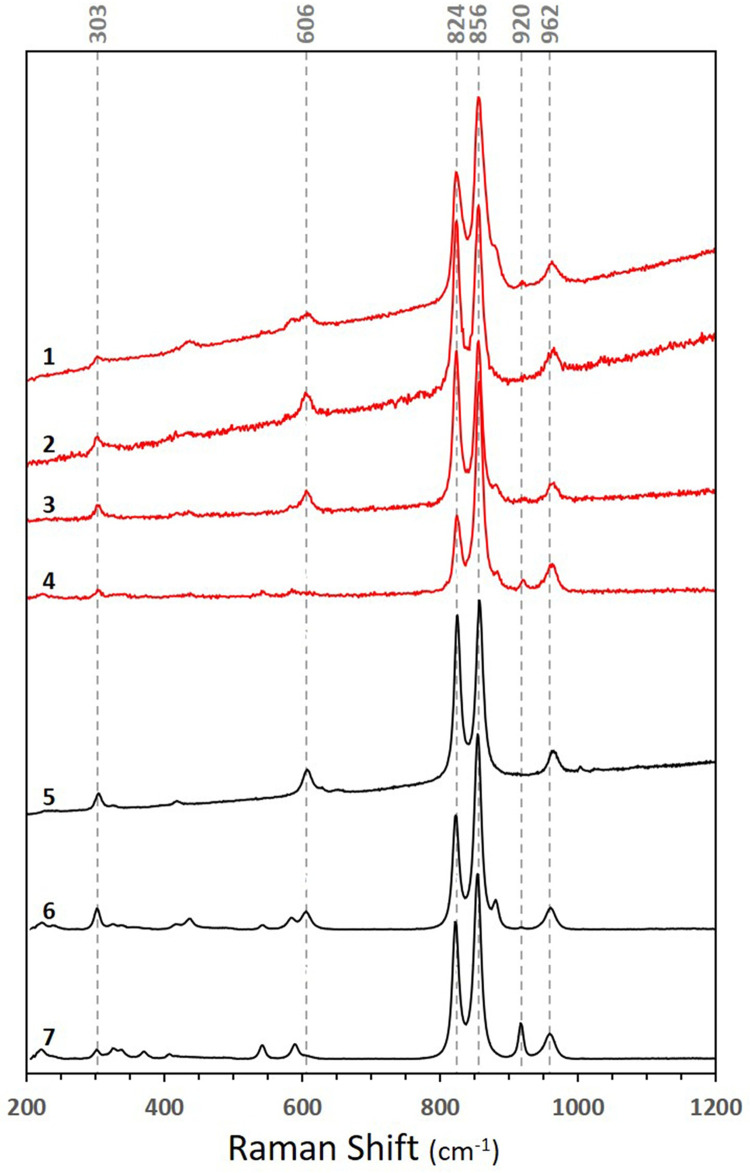
Raman spectra of four representative olivine beads (1–4) placed against three forsterite-rich olivine references. The references were acquired from https://rruff.info/, comprising olivine from San Carlos, Arizona, USA (5; RRUFFID = R040018), Zabargad Island, Egypt (6; RRUFFID = X050085), and East Africa (7; RRUFFID = X050081).

The Raman spectra collected from all beads are almost identical (Fig 5), consisting of a prominent doublet peak at 856 and 824 cm^-1^ and minor peaks at 962, 920, 606, and 303 cm^-1^. The doublet peak’s position indicates Mg-rich olivine compositions with Fo# of 90%–100% [72]. This conclusion was reinforced by the ion proportions and major element composition determined by the EPMA (Table 3) that found 0.993±0.004 (1σ) Si atoms and 2.002±0.008 Mg+Fe atoms per formula unit (4 oxygen atoms), corresponding with the chemical features of forsterite and fayalite (Mg_2_SiO_4_ and Fe_2_SiO_4_, respectively). Furthermore, Mg/Fe ratio suggests a composition of 89.5%–91.5% forsterite (Fo#). Minor elements in the olivine beads have limited composition as well, varying between Ni = 2870±120 ppm, Mn = 880±100, Ca = 450±90, Cr = 100±30, and Al = 70±20 ppm.

**Table 3:** Chemical composition of olivine beads.

Bead sample	Tsaf-Olivine1*	Tsaf-Olivine2	Tsaf-Olivine2	Tsaf-Olivine3	Tsaf-Olivine3	Tsaf-Olivine4	Tsaf-Olivine4	Tsaf-Olivine5	Tsaf-Olivine5	Tsaf-Olivine6	Tsaf-Olivine6	Tsaf-Olivine7	Tsaf-Olivine7	Tsaf-Olivine8	Tsaf-Olivine8
	a	a	b	a	b	a	b	a	b	a	b	a	b	a	b
Oxide wt.%															
SiO_2_	40.50	39.50	40.50	41.70	40.80	40.20	40.3	40.80	40.70	40.10	39.80	41.00	40.60	40.20	41.20
Al_2_O_3_	0.01	0.01	0.01	0.01	0.02	0.01	0.014	0.01	0.01	0.01	0.01	0.01	0.01	0.02	0.02
Cr_2_O_3_	0.01	0.01	0.02	0.02	0.01	0.02	0.023	0.01	0.01	0.01	0.01	0.01	0.01	0.02	0.02
FeO	8.50	9.80	9.50	8.80	8.80	10.30	10.3	8.40	8.50	9.70	9.70	8.60	8.70	9.30	10.00
MgO	50.40	48.60	48.90	50.90	50.50	49.00	49.00	49.70	49.90	48.50	48.60	50.40	50.30	49.70	50.30
CaO	0.04	0.07	0.07	0.06	0.06	0.09	0.08	0.06	0.07	0.05	0.05	0.05	0.05	0.07	0.07
MnO	0.09	0.12	0.11	0.10	0.10	0.13	0.12	0.11	0.10	0.13	0.13	0.11	0.11	0.11	0.12
NiO	0.37	0.33	0.35	0.38	0.37	0.37	0.36	0.39	0.38	0.36	0.34	0.38	0.37	0.37	0.36
Total	99.90	98.50	99.50	101.90	100.60	100.10	100.20	99.40	99.60	98.80	98.70	100.60	100.10	99.70	102.00
No. of ions per 4 oxygens															
Si	0.99	0.987	0.998	0.998	0.991	0.99	0.989	1.00	0.997	0.995	0.992	0.996	0.991	0.988	0.992
Al	0.0002	0.0004	0.0004	0.0004	0.0004	0.0004	0.0004	0.0003	0.0004	0.0003	0.0003	0.0003	0.0003	0.0006	0.0006
Cr	0.0002	0.0003	0.0003	0.0003	0.0002	0.0004	0.0005	0.0002	0.0002	0.0002	0.0002	0.0003	0.0002	0.0005	0.0004
Fe	0.175	0.205	0.197	0.176	0.179	0.211	0.212	0.171	0.173	0.202	0.202	0.175	0.177	0.191	0.201
Mg	1.835	1.81	1.795	1.816	1.828	1.797	1.797	1.817	1.821	1.795	1.804	1.822	1.83	1.821	1.803
Ca	0.001	0.002	0.002	0.002	0.002	0.002	0.002	0.002	0.002	0.001	0.001	0.001	0.001	0.002	0.002
Mn	0.002	0.002	0.002	0.002	0.002	0.003	0.003	0.002	0.002	0.003	0.003	0.002	0.002	0.002	0.003
Ni	0.007	0.007	0.007	0.007	0.007	0.007	0.007	0.008	0.008	0.007	0.007	0.007	0.007	0.007	0.007
Total	3.01	3.013	3.002	3.002	3.009	3.01	3.01	3.00	3.003	3.005	3.009	3.004	3.009	3.012	3.008
Fo (mol%)	91	90	90	91	91	90	89	91	91	90	90	91	91	91	90

*All analyses are single spots measurements.

We hypothesized above, on the grounds of crystal size, that the olivine beads of Tel Tsaf derive from mantle rocks. Now, our chemical analyses provide further support: the beads’ Fo#, Ni, and Mn contents are well within the ranges of mantle olivine, which are 89%–94%, 2200–3400 ppm, and 450–1050 ppm, respectively [73–75]. Moreover, Ca and Al concentrations in the beads are below the maximum values for these elements in mantle olivines (~650 and 150 ppm, respectively). They are also far below their values in other igneous olivines, usually above 1000 and 100 ppm, respectively [74,75]. Thus, in all likelihood, the olivine beads of Tel Tsaf originate in mantle rock fragments. On the Earth’s surface, such rocks occur in one of two ways: as xenoliths—where mantle fragments are incorporated in ascending magmas—or as ophiolites—exposed segments of oceanic crust and upper mantle.

## 5. Discussion

**Fig 6:**
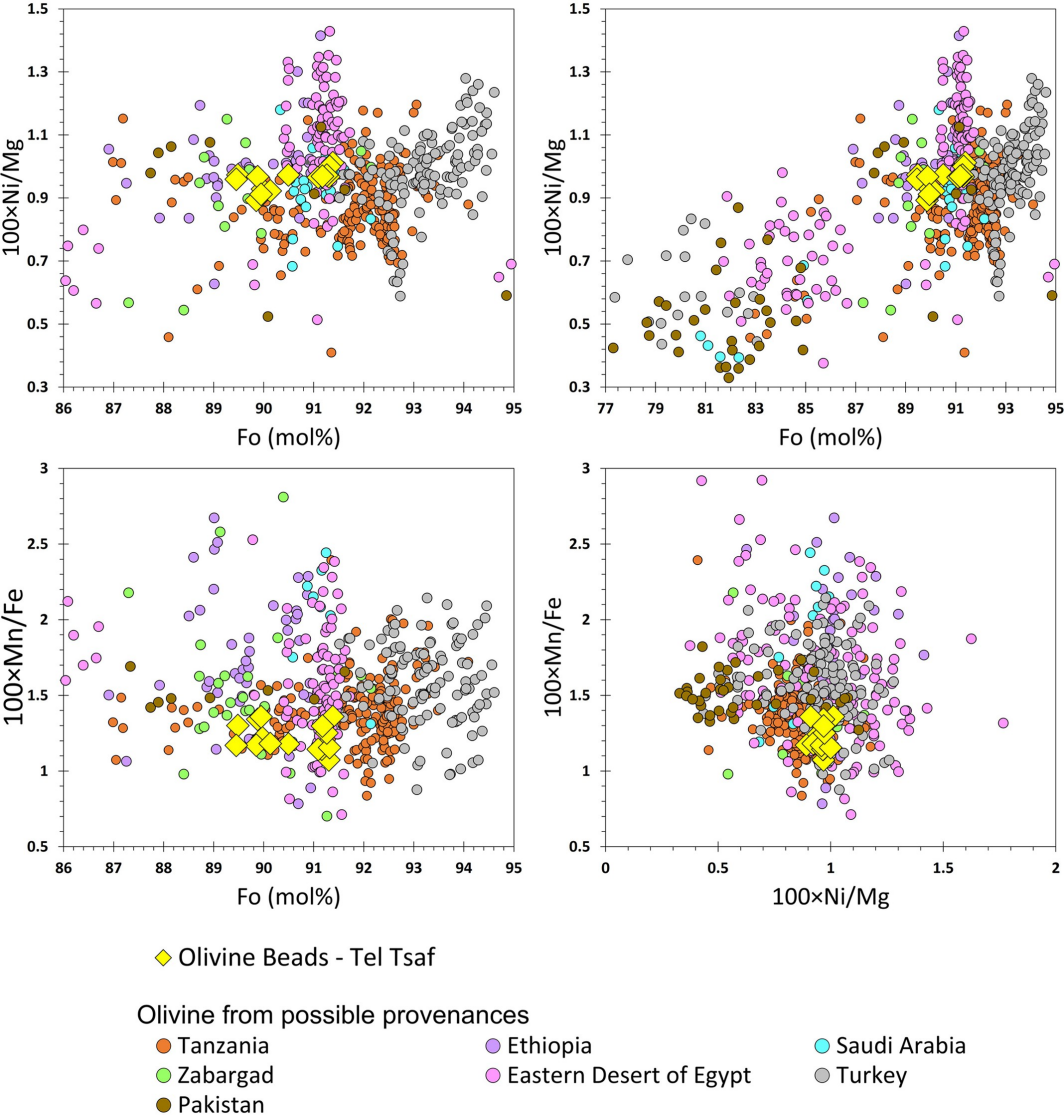
Chemical features of the Tel Tsaf olivine beads and possible olivine sources. (a) 100×Ni/Mg against Fo# spanning 86%–95%; (b) 100×Ni/Mg against Fo# spanning 77%–95%; (c) 100×Mn/Fe against Fo# spanning 86%–95%; (d) 100×Ni/Mg against 100×Mn/Fe. The data for the possible olivine origins were acquired from the following sources: For Turkey [29–31], for Pakistan [32–34], for Eastern Desert of Egypt [24], for Zabargad Island [21–23], for Saudi Arabia [25–28], for Ethiopia [35–37], and for Tanzania [38–40]. Several samples are not presented here; they include specimens from the Eastern Desert of Egypt with Fo>97% and Ni<2000 ppm and a few specimens from Turkey and Pakistan with Fo = 100.

**Fig 7:**
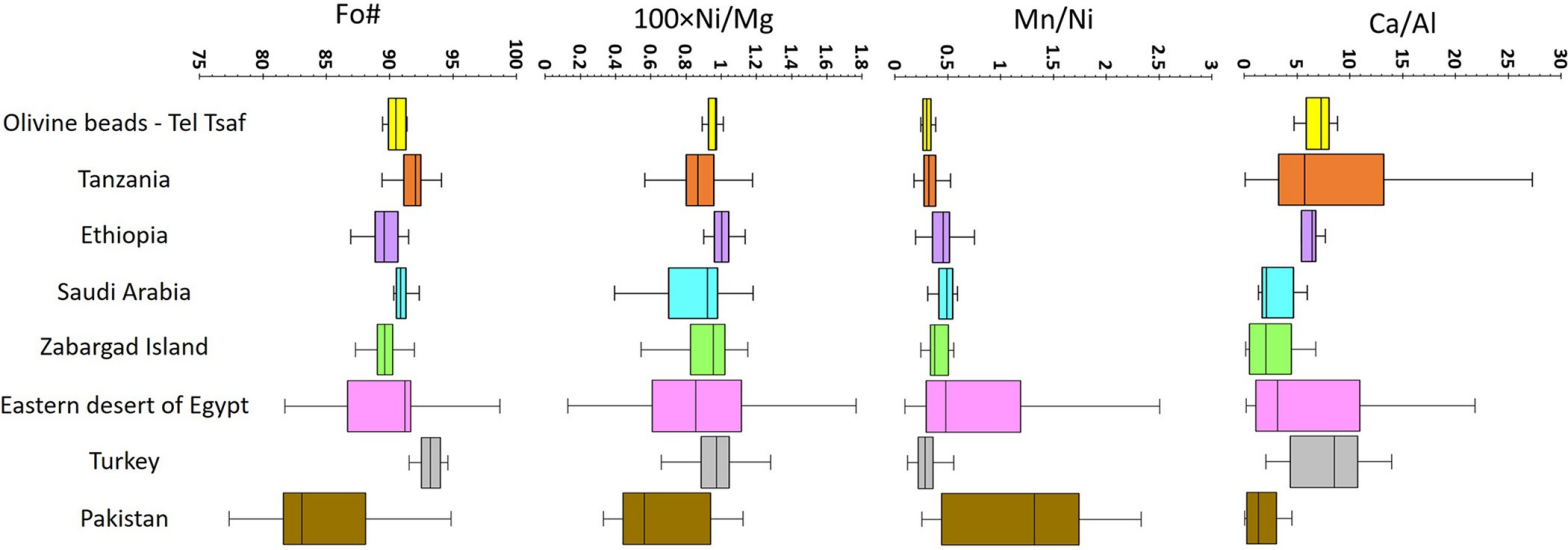
Box and whiskers plot for key chemical features of the Tel Tsaf olivine beads and possible olivine sources. Chemical features presented are Fo#, Ni/Mg, Mn/Ni, and Ca/Al compositions. The “whiskers” span the 1.5×interquartile range, while the boxes represent the median, the 25^th^, and 75^th^ percentiles (outliers excluded). The references for the data from the possible provenances are as in Fig 6.

The eight olivine beads from Middle Chalcolithic Tel Tsaf are the earliest specimens of their kind in the Near East and a rare incidence in the southern Levant. As noted above, six potential olivine sources have been identified: Turkey, Pakistan, Egypt’s Eastern Desert and Zabargad, Ethiopia, Tanzania, and Saudi Arabia. Of these, the Fo# of Turkey’s and most of Tanzania’s olivines are higher (92.5%–94.5% and 93.5%-93.5%, respectively; see Fig 7) than those of the beads from Tel Tsaf and, thus, are unlikely to have provided the raw crystals (Fig 6). Similarly, Pakistan can be set aside due to its olivine’s comparatively low Fo# between 77%–85% and Ni/Mg ratios. The statistical variations of the olivine populations’ chemical compositions further reinforce these observations (Fig 7). Mn/Ni and Ca/Al ratios clearly distinguish the olivines from Pakistan from those of Tel Tsaf, while Turkey’s and Tanzania’s olivines have too high Fo# to be viable candidates for the beads’ provenance. None of the remaining three potential sources (Saudi Arabia, Ethiopia, and Egypt) can be considered a substantially more probable source than the others. Nevertheless, it is notable that, statistically, the olivines from Ethiopia are closest to the beads from Tel Tsaf.

Either way, one of the most important observations is that the beads are chemically almost identical. This, in turn, suggests they derived from a single source and most probably arrived together at Tel Tsaf, possibly as a single chain or necklace. If indeed the Tel Tsaf olivine beads originate from one of the three potential sources mentioned above, long-distance trade was inevitably implicated (the Ethiopian plateau and the main Ethiopian rift area are over 2,000 km away). The beads had to travel via a long chain of hubs across far-flung and diffused systems of trade and exchange.

Raw materials and artifacts of long-distance trade are a familiar feature of the south Levantine Chalcolithic period [76–82]. The most striking example of this is the relatively widespread occurrence of Anatolian obsidian, especially during the Early and Middle Chalcolithic periods [61,77,83–87]. The eight olivine beads discussed here add another significant trade connection for the region, in general, and Tel Tsaf, in particular. As noted, the site’s long-distance trade appears to have been particularly intensive, indicated by a wide range of artifacts: a copper awl [60], Nilotic shells, beads, north Levantine tokens and figurines, Ubaid ceramics, and obsidian [11,44,52,88,89].

In all likelihood, the olivine beads constituted personal objects and were entwined with themes of value, desire, and social status. As a product, they boast high production value in terms of technology and distribution [90]. Furthermore, embodying distance, transportation, and far-reaching contacts conferred onto them high use-value.

The abundance of long-distance traded artifacts and substances at Tel Tsaf indicates that it accommodated a community capable of obtaining and managing surplus to foster economic and social connections. Moreover, along with the circulation of tangible goods, exchange networks also provide an infrastructure for the flow of intangible cultural assets communicated by artifacts that convey coded information, interpreted or read as ‘value’ or social/economic significance. In the case of the olivine beads, the transmitted coded information pertains to their value that derives from acknowledging their distant origin and the difficulty obtaining them.

However, modeling this multi-layered interaction system is nearly impossible due to numerous indeterminate agents and ambiguous factors. Nevertheless, we have good reason to presume that the value bestowed on exotic materials [91] is likely to have fed the motivation to obtain these beads. The properties of olivine (i.e., color, translucence, and reflectivity) coupled with its distant origins might have been instrumental for its far-flung circulation. The olivine beads’ significance should be appreciated not only for their rare exoticism but also as indicators of Tel Tsaf’s constitution as a pan-regional hub that engaged in (and probably controlled) superregional exchange during the late sixth–early fifth millennia cal. BC.
